# Spontaneous Bilateral Tubal Ectopic Pregnancy in a Low-Risk Patient: A Case Report with Implications for Preoperative Patient Counseling

**DOI:** 10.1155/2021/5588869

**Published:** 2021-06-19

**Authors:** Josephine Eva Gathura, Amro Elfeky, Rodney McLaren, David Herzog, Richard Grazi

**Affiliations:** Department of Obstetrics and Gynecology, Maimonides Medical Center, Brooklyn, New York, USA

## Abstract

Bilateral tubal ectopic pregnancies (BTP) are the rarest form of ectopic pregnancy. They are difficult to diagnose preoperatively, and an evidence-based guideline for management does not exist. In this report, we discuss a 35-year-old patient who presented with suspected right tubal ectopic pregnancy. BTP was diagnosed intraoperatively, and a laparoscopic bilateral salpingectomy was performed without complication. The diagnosis was subsequently confirmed by pathology. This case highlights the importance of patient counseling and comprehensive preoperative planning. Due to the poor presurgical diagnosis of BTP, patient counseling should include the possibility of BTP, appropriate options for management, and potential loss of fertility following treatment. In addition, all cases of suspected ectopic pregnancy necessitate a thorough preoperative investigation of bilateral adnexa and intraoperative inspection of the pelvis.

## 1. Introduction

Ectopic pregnancies account for approximately two percent of all reported pregnancies and are most commonly found in a unilateral fallopian tube [1]. Bilateral tubal ectopic pregnancy (BTP) is extremely rare, commonly followed by assisted reproductive technology (ART). The estimated incidence of BTP is 1 in 725 to 1 in 1580 of all ectopic pregnancies [2]. This corresponds to an occurrence of 1 per 200,000 live births [3]. The true incidence of BTP, however, may be higher as many cases can go unrecognized secondary to spontaneous resolution or successful medical management of presumed unilateral tubal ectopic pregnancies. BTP is clinically indistinguishable from a unilateral tubal ectopic pregnancy, and most cases are incidentally diagnosed at the time of surgery. An evidence-based guideline for management does not exist.

## 2. Case Report

**Figure 1:**
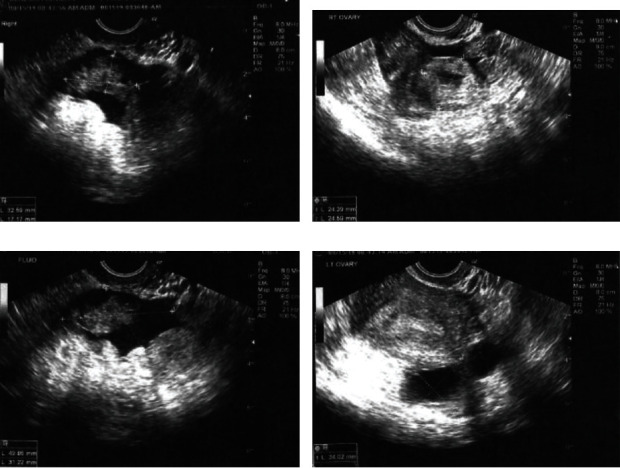
Transvaginal ultrasound findings. (a) Right adnexa. (b) Right ovary. (c) Pelvic fluid. (d) Left ovary.

A 35-year-old woman G4P0030 at 5 weeks and 6 days based on the last menstrual period initially presented to a reproductive endocrinologist (REI) for recurrent pregnancy loss. Past medical history was significant for three prior spontaneous abortions, the last of which was noted to be a pregnancy of unknown location, and was managed expectantly with spontaneous resolution. Past surgical history was unremarkable. Prior to starting any intervention, the patient was found to be pregnant. The beta-human chorionic gonadotropin (*β*-hCG) trend revealed the following values: 776 mIU/mL (day 1), 1203 mIU/mL (day 3), 2211 mIU/mL (day 7), and 2669 mIU/mL (day 9). A transvaginal ultrasound performed on day 9 showed a lack of an identifiable intrauterine pregnancy (IUP), a right adnexal mass measuring 33 × 17 mm, and a moderate amount of free fluid in the pelvis. No left adnexal mass was reported (Figure 1).

The patient was subsequently referred to the emergency room for complaints of moderate right lower quadrant pain in the setting of a pregnancy of unknown location with abnormally rising *β*-hCG. In the emergency room, the patient was hemodynamically stable with blood pressures 120 s/80 s mmHg and heart rate 70 s beats per minute. On physical exam, the abdomen was soft, nondistended, and nontender without rebound or guarding. *β*-hCG was obtained and found to be 5454 mIU/mL. The repeat transvaginal ultrasound revealed the same findings as the ultrasound performed at the REI office. The patient was made aware of the concern for a ruptured ectopic pregnancy due to physical exam findings and free fluid on transvaginal ultrasound evaluation, without evidence of an IUP. Surgical management of ectopic pregnancy was advised, and consent was obtained for a diagnostic laparoscopy and possible salpingectomy or salpingostomy.

**Figure 2:**
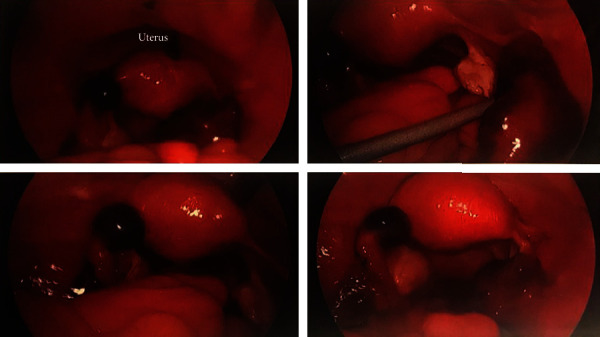
Intraoperative photos of the uterus and bilateral adnexal masses.

Intraoperatively, the uterus and ovaries were within normal limits. Approximately 100 mL of dark blood was present and evacuated from the cul de sac. A large, right ampullary ectopic pregnancy with damaged fimbria and fallopian tube was appreciated. The isthmic portion of the left fallopian tube was noted to have a blue-purple bulge suspicious for ectopic pregnancy versus a hemorrhagic or necrotic process (Figure 2). Due to the patient's history of infertility and desire to undergo in vitro fertilization, the REI and the family were contacted intraoperatively to discuss the findings of suspected BTP and to formulate a surgical plan that aligned with the patient's goals. The decision for definitive management was made, and bilateral salpingectomy was performed without complication.

The final diagnosis of bilateral tubal ectopic pregnancy was confirmed on pathology. The pathology report demonstrated chorionic villi within the right fallopian tube consistent with right intratubal ectopic pregnancy. The left fallopian tube demonstrated focal decidua and few trophoblast-like cells, which were positive for placental alkaline phosphatase suggestive of left tubal ectopic pregnancy. The patient's postoperative course was uneventful, and the *β*-hCG decreased appropriately following the procedure.

## 3. Discussion

BTP is a rare form of ectopic pregnancy, and BTP in the absence of preceding use of ART, as seen in this case, is considered to be the rarest form. The clinical presentation of unilateral versus bilateral tubal ectopic pregnancy is indistinguishable. Patients will often present with the classic triad of symptoms of amenorrhea, vaginal bleeding, and abdominal pain. Further investigation may reveal an inappropriately rising *β*-hCG level with or without sonographic evidence of tubal ectopic pregnancy. Ultrasonography is capable of diagnosing ectopic pregnancy when a gestational sac with a yolk sac or embryo is present within the tube, with a positive predictive value of 80 percent [1]. Most ectopic pregnancies do not advance to reach this stage, and the evaluation of the contralateral tube does not guarantee proper identification of BTP. Thus, due to the overlap of symptoms and the limitation of ultrasonography in the diagnosis of ectopic pregnancy, BTP is oftentimes diagnosed at the time of surgery rather than accurate preoperative diagnosis. To date, more than 200 cases have been reported in the literature, with only three cases of BTP diagnosed preoperatively by ultrasound [3–5]. There is a lack of data to support the use of additional imaging modalities for the diagnosis of BTP. Consequently, it is important to consider BTP in the preoperative planning period when investigating the adnexa and providing preoperative counseling, especially with patients who desire future fertility. The patient in our reported case would have had the opportunity to take part in the surgical planning after being aware of the possibility of BTP and the potential outcomes, which included bilateral salpingectomy. This would have also eliminated the need to contact the REI specialist and family intraoperatively to formulate a surgical plan.

Typically, for patients with a suspected unilateral tubal ectopic pregnancy to be considered for medical management, they must meet eligibility criteria for methotrexate administration and be monitored closely with serial *β*-hCG until resolution of pregnancy is confirmed. However, it is unknown whether medical management is an acceptable option for BTP because ultrasonography remains an unreliable method for the diagnosis of BTP. Furthermore, no studies address the dosing regimen and efficacy of methotrexate when treating BTP. Accurate diagnosis is an essential prerequisite to medical management, as failed medical management for BTP has been reported after the administration of a single dose injection of methotrexate for presumed unilateral tubal ectopic pregnancy [2]. To date, only one case of a spontaneous BTP was reported that was diagnosed by ultrasonography and successfully treated by two consecutive intratubal methotrexate injections under vaginal ultrasonographic guidance without complications [4]. The patient discussed in our case, however, was not eligible for medical management due to clinical signs consistent with ruptured ectopic pregnancy.

Patients who are hemodynamically unstable or symptomatic, have findings concerning for rupture, or have any contraindication to medical management should be managed surgically. It is important to note that surgical intervention does not guarantee the detection of BTP. As such, a thorough inspection of the pelvis is required to avoid missed intraoperative diagnosis which may lead to unnecessary repetitive procedures and rupture of the undetected ectopic pregnancy [2].

The question remains whether salpingostomy is a conservative surgical option for eligible patients with BTP given the possible risk of recurrent ectopic pregnancy and the possible need for additional medical or surgical management in the event of persistent ectopic pregnancy, while considering fertility preservation as compared to definitive radical surgical treatment with bilateral salpingectomy. There are two reported cases of intraoperatively diagnosed BTP treated with bilateral salpingostomy, one of which required a dose of intramuscular methotrexate for persistent ectopic pregnancy [2, 6]. There are a few reported cases of intrauterine conception following surgical management of BTP with unilateral salpingectomy and contralateral salpingostomy, the most recent of which was reported in 2013 [2, 4]. Nonetheless, the majority of BTP cases undergo bilateral salpingectomy, as did our patient.

In this case, our patient will need ART to achieve pregnancy. To date, the IVF success rate is approximately 27% [7]. For patients who undergo unilateral salpingostomy or unilateral salpingectomy with a contralateral healthy tube, the spontaneous pregnancy rate is approximately 56-60% [8]. This should be considered alongside accessibility of fertility services when pursuing surgical management. Fertility services are readily available in the United States. However, access to ART can be limited and should not be assumed to be a viable option for all patients [9].

## 4. Conclusion

**Figure 3:**
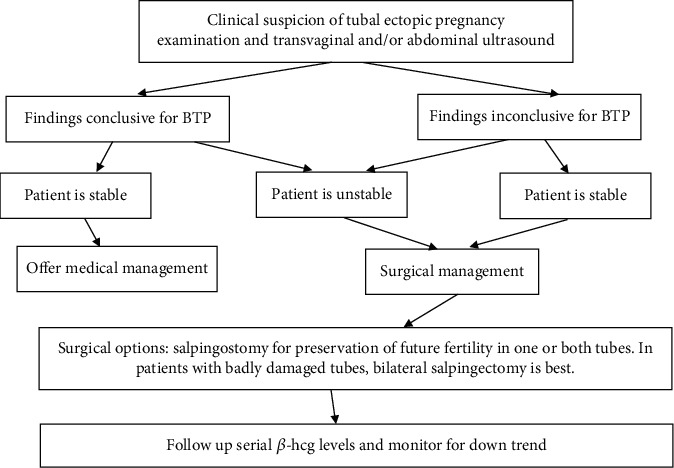
Algorithm for management of suspected bilateral tubal ectopic pregnancy (BTP). Modified from Jena SK, et al. Bilateral Simultaneous Tubal Ectopic Pregnancy: A Case Report, Review of Literature and a Proposed Management Algorithm. J Clin Diagn Res. 2016; 10(3).

Ectopic pregnancy still remains the leading cause of maternal mortality in the first trimester, accounting for 4 to 10 percent of all pregnancy-related deaths, making timely diagnosis and appropriate management of the utmost importance. [3]. An algorithm for the management of suspected BTP has been proposed by Jena et al. to address this concern and decrease the chances of missed or delayed diagnosis (Figure 3) [2]. Ideally, an evidence-based protocol would guide the management of BTP; however, given the low incidence and poor presurgical diagnosis of BTP, such a protocol has to be extrapolated from current case reports in the literature and individualized for each case.

Although rare, BTP can be a life-threatening condition if misdiagnosed and should be considered in all cases of suspected ectopic pregnancy. The case described highlights the importance of patient counseling and comprehensive preoperative planning. Patient counseling should include the possibility of BTP, appropriate options for management, and the potential loss of fertility following surgical management. This approach promotes both shared-decision making and preparedness which are needed to provide patient-centered and comprehensive care. Lastly, a thorough preoperative investigation of the bilateral adnexa and inspection of the pelvis intraoperatively may mitigate the risk of persistent ectopic pregnancy and resulting sequelae.
